# A role for an HTLV-1 vaccine?

**DOI:** 10.3389/fimmu.2022.953650

**Published:** 2022-09-08

**Authors:** Lee Ratner

**Affiliations:** Division of Oncology, Department of Medicine, Washington University School of Medicine, St Louis, MO, United States

**Keywords:** HTLV 1, TAX, envelope (E), glycoprotein (GP), vaccine, neutralizing abs, cytotoxic T cell

## Abstract

HTLV-1 is a global infection with 5-20 million infected individuals. Although only a minority of infected individuals develop myelopathy, lymphoproliferative malignancy, or inflammatory disorders, infection is associated with immunosuppression and shorter survival. Transmission of HTLV-1 is through contaminated blood or needles, mother-to-child exposure through breast-feeding, and sexual intercourse. HTLV-1 is a delta retrovirus that expresses immunogenic Gag, Envelope, TAX, and Hbz proteins. Neutralizing antibodies have been identified directed against the surface envelope protein, and cytotoxic T-cell epitopes within TAX have been characterized. Thus far, there have been few investigations of vaccines directed against each of these proteins, with limited responses, thus far. However, with new technologies developed in the last few years, a renewed investigation is warranted in search for a safe and effective HTLV-1 vaccine.

## HTLV-1

**Figure 1:**
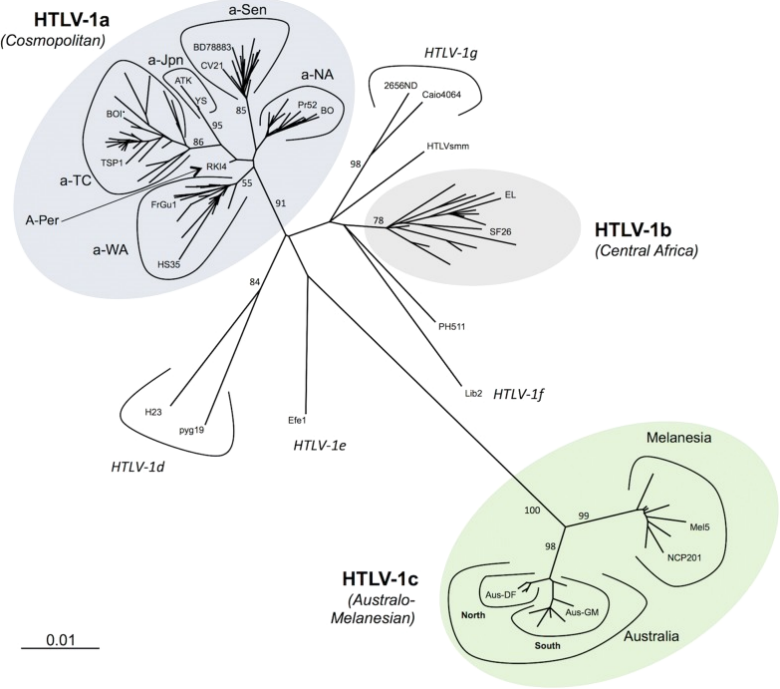
Phylogenetic representation of the HTLV-1 genotypes and subgroups. An alignment of complete LTR sequences (774 nucleotides long) from 178 HTLV-1 strains was obtained. The unrooted phylogenetic tree was generated with the neighbor-joining method using the GTR model (gamma=0.50). Branch lengths are drawn to scale, with the bar indicating 0.01 nucleotide replacements per site. Numbers on each node indicate the percentage of bootstrap samples (of 1000 replicates). HTLV-1 genotypes (a-g) and subgroups with HTLV-1a and HTLV-1c are presented. Published with permission from the author and journal (4).

HTLV-1 is prevalent in many parts of the world, including Central & South America, Caribbean Islands, Africa, northeast Iran, southern Japan, Melanesia, Australia, where endemic rates are 5-10%, but in some isolated communities endemic rates as high as 50% have been identified (1, 2). However, several large and highly populated regions in India and North and East Africa have not been screened. In the US, the prevalence of HTLV infection is 0.1-0.2% (3). HTLV-1 strains are highly conserved with <2% overall divergence for cosmopolitan strains (HTLV-1a) from most areas of the world, with up to 10% divergence with strains from Australia and Melanesia (HTLV-1c)(**Figure 1**) (4). Strains from central Africa (HTLV-1b, d-g) are somewhat more divergent than the HTLV-1a strains.

Diseases caused by HTLV-1 include an aggressive CD4+ lymphoproliferative malignancy, designated adult T-cell leukemia lymphoma (ATLL) and spastic paraparesis, known as HTLV-1 myelopathy (HAM) or tropical spastic paraparesis (TSP) (5). Although, only 5-10% of infected individuals develop these disorders, HTLV-1 is associated with many other clinical inflammatory disorders, immunosuppression, and shortened survival (6). Sequence differences in the virus do not correlate with disease development (7). HTLV-1 causes a lifelong persistent infection, which is never truly silent (8). This led to an increased focus by the WHO on HTLV epidemiology and prevention strategies, which included a recommendation to develop a global strategy for the elimination of HTLV-1 (9).

Transmission of HTLV-1 is through contaminated blood or needles, sexual intercourse, and breast feeding (10). Rarely, HTLV is transmitted through ritual scarification practices (11). Zoonotic transmission through severe bites from simian T-cell leukemia virus type 1 infected non-human primates have also occurred among hunters in central Africa (12). Transmission of HTLV-1 from mother-to-child can be reduced by screening and education, which is a nationwide strategy in Japan (13). Horizontal transmission of HTLV-1, by sexual intercourse or blood transfusion is also preventable (14). An additional emerging concern is HTLV-1 infection upon organ transplantation (15). Attempts to facilitate screening remain to be developed.

After transmission, a balance between virus replication, expansion of infected cells, and immune response to the virus leads to the establishment of a proviral load “set point” after HTLV-1 acquisition. However, there have been few studies of acute HTLV-1 infection aimed at assessing the determinants and kinetics of the set point. In a study of three individuals who acquired HTLV-1 infection after organ transplantation, the proviral load set point was reached within 6 weeks (15). Thus, therapeutic approaches to infection prophylaxis have a limited time window in which to act. Nevertheless, it remains unclear whether transmission through other routes establishes the proviral set point with similar kinetics.

Cellular transfer of virus occurs more commonly *via* cell-to-cell contacts than *via* free virus particles (16). Two types of cell-cell contacts have been described to be critical for HTLV-1 transmission, tight junctions and cellular conduits (17). Non-exclusive mechanisms of virus transmission at cell-cell contacts include polarized budding into synaptic clefts and cell surface transfer of viral biofilms at virological synapses (18, 19). In contrast to CD4+ T-cells, dendritic cells can be infected with cell-free virus and, to a greater extent, *via* viral biofilms (20).

HTLV-1 is a member of the δ retrovirus family, which also includes HTLV-2 and bovine leukemia virus (BLV) (21). HTLV-2 is not clearly associated with disease, whereas BLV is a cause of B cell lymphoproliferative disorders in cattle. HTLV-1 encodes classical retrovirus structural proteins from group-specific antigen (*gag*) and envelope (*env*) genes, and enzymes from the protease (*pr*) and polymerase (*pol*) genes that encode the viral protease, reverse transcriptase (RT), and integrase (IN). Virus infection is mediated by a receptor complex on the cell surface and the virus particle is taken into cells by membrane fusion. Virus uncoating activates RT to copy the two copies of the plus-strand viral RNA genome into a dsDNA copy that is integrated into cellular DNA by the viral IN. Transcription is mediated by cellular RNA polymerase II, and viral RNAs may be spliced or unspliced prior to export from the nucleus. Translation produces viral proteins, including the Gag, Gag-Pr, and Gag-Pr-Pol polyprotein precursors which are processed into individual components during virus budding. Envelope is synthesized and processed in the reticuloendothelial-Golgi system, transported to the plasma membrane, and incorporated into the budding virus. Regulatory proteins, are encoded from multiple spliced RNAs and include the transactivator protein, TAX, the regulator of splicing and nuclear export, REX, the helix zipper protein, HBZ, as well as proteins designated p12, p27, and p30 that are presumed to regulate virus replication and/or pathogenesis.

HTLV-1 encodes two oncoproteins, TAX and HBZ (22–24). TAX promotes cytoplasmic signaling through various receptors and causes abnormal cell cycle regulation, genetic instability, and inhibition of DNA repair and apoptosis (25). HBZ counteracts the functions of TAX promoting a persistent latent infection (26). HBZ regulates signaling pathways important for inflammation, transcription, apoptosis, autophagy, histone methylation, and T-cell differentiation (27).

## HTLV-1 envelope glycoprotein

**Figure 2:**
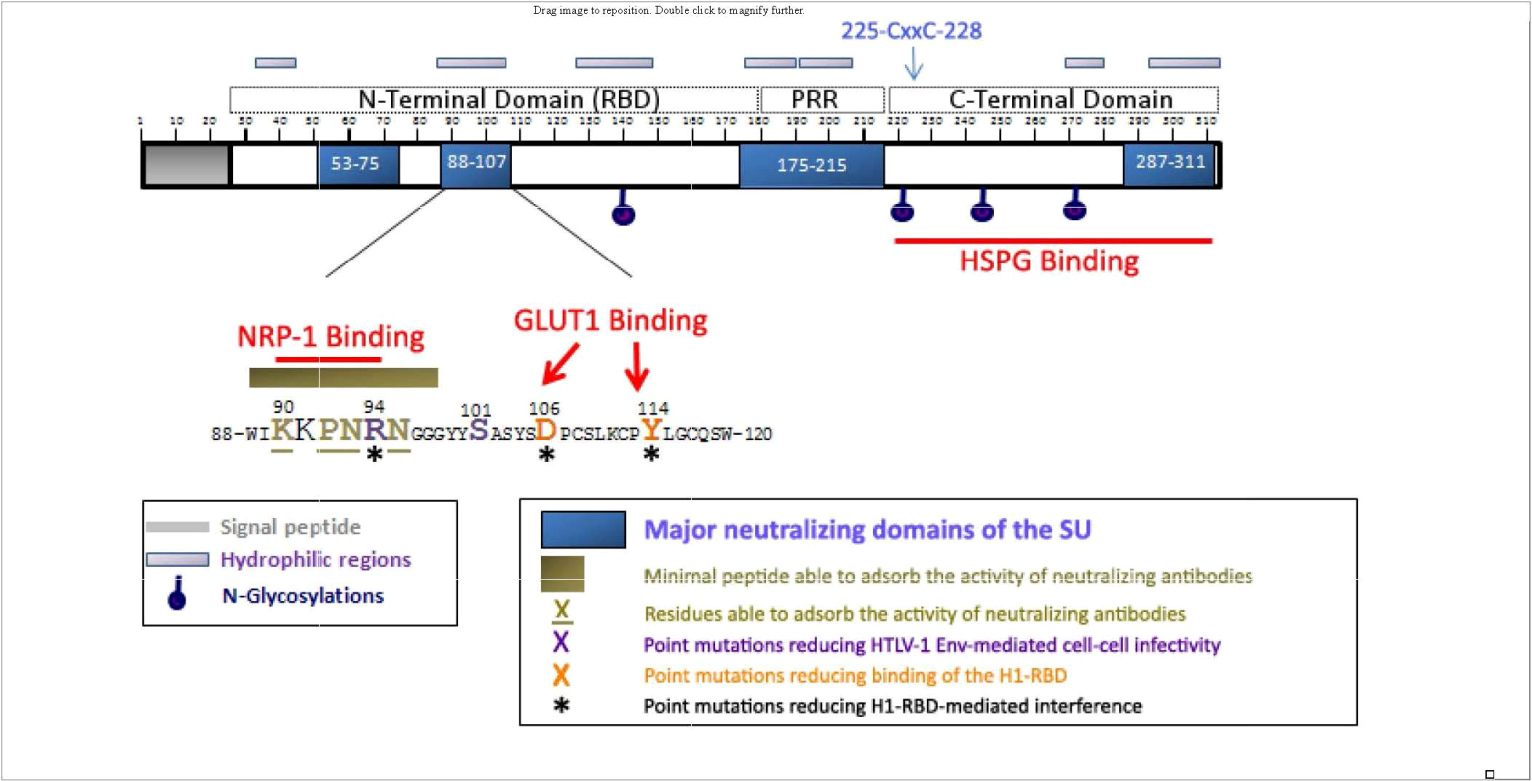
Localization of the neutralizing regions and the domains and residues involved in HSPGs, NRP-1, and GLUT-1 binding within the HTLV-1 SU protein. The 90-94 motif identified as critical for direct NRP-1 binding corresponds to a minimal neutralizing epitope, and contains the R94 residue required for HTLV-1 particle infectivity. R94, as well as D106 and Y114 that mediate binding of the H1-RBD to target cells are required for H1-RBD-mediated receptor interference. The C-terminal domain of the SU contains the determinants for HSPG binding. Published with permission from the author and the journal (28).

The HTLV-1 *env* gene encodes the gp62, 488 amino acid envelope precursor glycoprotein which is cleaved by furin-like enzymes into a 20 amino acid (AA) signal peptide, a 292 amino acid gp45 surface envelope protein (SU) and a 176 amino acid gp21 transmembrane envelope protein (TM). SU has four asparagine (N)-linked glycosylation sites (AA 140, 222, 244, and 272; **Figure 2**), and TM has a single N-linked glycosylation site (AA 404), a disulfide bond (AA 393-400), and a S-palmitylated cysteine residue (AA 462). TM includes the fusion peptide (AA 313-333), and two coiled coil, heptad repeat (HR) domains (AA 341-387 and 397-429).

SU mediates infection by binding to cellular entry factors heparin sulfate proteoglycans (HSPG), glucose transporter 1 (GLUT-1), and neuropilin-1 (NRP-1) (28). Subsequent conformational changes include isomerization of a SU-TM intersubunit disulfide result in fusion of the viral and cellular membranes (29). Although the crystal structure for SU has not yet been determined, it has been proposed to contain two separate folding domains separated by a proline-rich linker peptide (PRR; **Figure 2**) (30). The N-terminal region, designated the receptor-binding domain (RBD) is necessary and sufficient for binding to GLUT-1. The C-terminal domain includes the binding site for HSPG.

Retroviral envelope proteins, like those of coronaviruses, arenaviruses, filoviruses, pneumoviruses, and orthomyxoviruses are class I fusion proteins (31). They are all type I single-pass trans-membrane proteins that form non-covalently linked homotrimers in their pre- and post-fusion conformations. The structural hallmark of class I fusion proteins is the parallel trimeric α-helical coiled-coil in the post-fusion C-terminal subunit (TM in the case of retroviruses). This long α-helix of the coiled coil contains the HR with non-polar amino acids at positions 1 and 4 of the repeats. The C-terminal portion of the α-helix runs antiparallel to the N-terminal portion along the grooves of the coiled-coil to complete the post-fusion hairpin, thereby making a trimeric postfusion six-helix bundle. Detailed structures are available for many of these proteins in each conformation. A common feature of these proteins is their dynamic conformational changes, presenting open and closed forms in equilibrium (32). There is a large body of research indicating that it is the closed form that is recognized by the known broadly neutralizing antibodies, whereas the epitopes exposed in the open form which do not bind these antibodies

## Neutralizing anti-HTLV-1 antibodies

Polyclonal and monoclonal antibodies to HTLV-I envelope proteins, including SU, have been demonstrated to neutralize HTLV-I infection (33–36). Neutralizing antibodies were observed in HTLV-1 infected individuals, which can prevent infection (37–40). These antibodies may interfere with receptor or coreceptor recruitment, or prevent receptor-induced changes in SU conformation that are required to activate the fusogenic properties of envelope (41). Major neutralizing domains of SU have been mapped to 4 domains (AA 53-75, 88-107, 175-215, and 287-311, **Figure 2**) (35, 36, 42). Efficacy of neutralizing antibodies was demonstrated by passive transfer of anti-envelope antibodies which block blood-borne or milk-borne HTLV-1 infection of rabbits (43–47)

## HTLV-1-specific cellular immunity

HTLV-1 infection elicits a strong CTL response (48). The frequency of HTLV-1-specific CTLs may be very high with up to 10% of circulating CD8+ T cells recognizing a single immunodominant CTL antigen target, TAX (49). The frequency of HTLV-1-specific CTLs is correlated with the HTLV-1 proviral load (50). This raised the hypothesis that HTLV-1-specific CTLs may fail to eradicate the virus, and may contribute to the inflammatory tissue damage with disease. presentation However, there is also evidence that CTL responses to HTLV-1 may be protective. Higher expression of CTL effector proteins is correlated with lower proviral load (51). In addition, the immunodominant TAX protein is subject to positive selection *in vivo* (52). Envelope-specific CTLs have also been detected, but are present at low frequencies (53, 54). T-cell epitopes within envelope in HLA-A2-positive individuals were mapped to residues 175-183, 182-190, 239-247, 395-403, and 442-450 (55, 56). The lytic efficiency of the CD8+ T cell response, as measured by the fraction of HTLV-1-expressing cells eliminated per CD8+ cell per day, was found to be inversely correlated with both the proviral load and the rate of spontaneous proviral expression (57). However, ATLL patients have weak CTL responses to HTLV-1 antigens (58).

## Vaccination against HTLV-1

Preventative vaccines are highly effective against a wide range of viral diseases, including cancer viruses, hepatitis B virus and human papilloma virus (59). There has also been a resurgence in therapeutic cancer vaccines (60), including malignancies caused by viruses (61), as well as non-viral cancers associated with production of neoantigens (62). However, most efforts focused on a vaccines for retroviruses have been concerned with HIV, and with very limited success, thus far (63). Nevertheless, an inactivated viral vaccine for feline leukemia virus has been developed that provides protection against heterologous strain infection (64). The vaccine did not prevent infection, but it did induce an antibody response and results in proviral loads more than 100-fold lower than challenged but non-vaccinated cats, that were detectable for a shorter time interval. A recombinant feline leukemia virus vaccine (PureVAX) is also commercially available, which utilizes a canary pox vector to express a mutated envelope, gag, and a truncated polymerase protein, resulting in 93% efficacy (65)

Several studies have evaluated vaccines against BLV (66). Inactivated BLV, crude lysates from persistently infected cell lines, peptide antigens, poxvirus-expressed BLV antigens, or DNA vaccines led to partial protection, with declining neutralizing antibody titers and poor stimulation of CTL responses. An attenuated BLV vaccine was made with deletions in TAX and the antisense accessory (G4) genes, resulting in a virus capable of very low levels of infectivity and replication, which is commercially available (67).

Kazanji et al. tested a chimeric peptide vaccine composed of B- and T-cell epitopes of HTLV-1 (68). They identified high titer antibodies and a high frequency of interferon γ-expressing cells against the envelope and TAX immunogens, but not against individual TAX peptides. After challenge, partial protection was achieved as evidenced by lower proviral loads in immunized compared to control animals. Studies with peptide immunization had previously been performed in rabbits by Takehara et al. (43). In that case, rabbits were vaccinated with a peptide corresponding to Env amino acids 175-196. Although pre-challenge sera from these rabbits showed high titers of anti-HTLV-1 Env antibodies, after challenge, virus was recovered from all rabbits.

A highly attenuated poxvirus expressing the entire HTLV-1 envelope protein was used to immunize New Zealand white (NZW) rabbits (69). The animals were protected from HTLV-1 infection, but not protected upon re-challenge 5 months later with 10- to 100-fold greater infectious virus load. It was unclear whether the lack of protection to re-challenge was a result of being overwhelmed with a large dose of virus, or due to the presence of a viral variant that evaded the immune response. Hokada et al. also inoculated rabbits with a recombinant vaccinia virus carrying the HTLV-1 envelope gene (70). This vaccine elicited anti-envelope and antibodies, but neutralizing antibodies were not detected. In addition, this vaccine did not prevent infection.

Nakamura et al. immunized 4 cynomolgus monkeys with 4-6 doses of *E coli*-produced HTLV-1 envelope protein (71). These animals were protected against infection, when challenged with a live HTLV-1 producing cell line, MT2. However, 2 monkeys inoculated with fewer doses did not produce antibodies that block HTLV-1 induced syncytium formation and were not protected against challenge with MT2 cells.

Ibuki et al. inoculated 2 cynomolgus monkeys with a recombinant vaccinia virus expressing the HTLV-1 envelope, and elicited neutralizing antibodies, and these animals were protected against infection (72). Kazanji et al. tested several HTLV-1 vaccines in squirrel monkeys (73). These included attenutated vaccinia virus-derived HTLV-1 *env* and/or *gag* expression vectors. However, after 3 inoculations, only one of three animals was protected against infection. Since naked DNA has also been used to induce neutralizing antibodies against HTLV-1 envelope glycoproteins in mice (74, 75), Kazanji et al. also incorporated this reagent in their studies (73). Priming animals with an *env* DNA vaccine followed by the recombinant vaccinia virus expressing *env* and *gag* resulted in protection of all three inoculated animals.

Kabirit et al. synthesized a multi-epitope chimeric protein with TAX, Env, and Gag immunodominant epitopes encapsulated in biodegradation poly(D,L-lactide-co-glycolide) nanoparticles (76). This preparation elicited antibody and cytokine responses in mice, but efficacy against infection was not reported. Humoral and cell-mediated immune response against the HTLV-1 envelope were detected in the protected animals.

A recombinant HTLV-1 glycoprotein protein vaccine was made against the HTLV-1c glycoprotein (77). This subunit vaccine utilized a molecular trimerization domain clamp to stabilize the prefusion conformation of the glycoprotein (78). This approach was previously used to stabilize influenza A hemagglutinins, using HIV-1-derived heptad regions. For the HTLV-1c envelope vaccine, the clamp was modified to negate production of anti-HIV-1 envelope antibodies. Use of several different adjuvants resulted in strong antigen-specific responses in mice.

MHC-I-bound HTLV-1 peptides have been identified which give rise to HTLV-1-specific CTLs *in vivo* (79). A therapeutic vaccine to activate TAX-specific CTLs was developed using TAX peptide-pulsed dendritic cells, resulting in favorable clinical outcomes in three ATLL patients (80, 81). These investigators also demonstrated that dendritic cells from peripheral blood mononuclear cells of a chronic ATLL patient evoked TAX-specific CTL-responses (82). However, since half of all ATLL patients lose the ability to express TAX, this approach may have limited utility (83). MacNamara et al. quantified the contribution of all HLA class I alleles to host protection against infection with HTLV-1 (84). They concluded that CD8+ cell response to HBZ are most effective. HTLV-1 carriers who had MHC class 1 alleles which could preferentially bind and present epitopes from HBZ were more likely to have low proviral loads, and less likely to develop disease than carriers who had MHC alleles which weakly bound HBZ peptides. Vaccination with a recombinant vaccinia virus expressing HBZ resulted in CTLs with anti-lymphoma effects in HBZ transgenic mice (85). In addition, this vaccine produced HTLV-1-specific T cell responses in infected rhesus monkeys (85).

## RNA vaccines

Messenger RNA (mRNA)-based vaccines hold the promise to revolutionize the infectious disease prevention field by addressing current manufacturing challenges and offering novel vaccine compositions (86). Critical quality attributes are high efficiency of expression with a 5’cap, 5’untranslated region of optimal length with key regulatory elements, codon optimization, 3’poly-A tail length appropriate for translation, and lack of impurities that induce inflammatory cytokines and reduce expression. The use of lipid nanoparticle (LNP) formulations stabilize the mRNA and facilitate cellular uptake. As of 2020, 12 clinical trials for mRNA-based infectious disease vaccines were completed for infectious agents including respiratory syncytia virus, rabies, chikungunya, zika, parainfluenza, influenza, and cytomegaloviruses. The recent success of coronavirus-19 mRNA vaccines has re-energized the field (87). These vaccines, based on a “universal” LNP delivery system, have proven tolerable and highly efficacious. Challenges remaining include thermal instability of the mRNA cargo, further optimization of the nanoscale delivery platform to produce target-specific immunoactivation and prolong the duration of the effect, achieving a “one-shot” approach, achieving low cost for low- to middle-income countries, lack of clarity about the longevity and type of immunoprotection offered, and hypersensitivity reactions. This approach is worthy of investigation for HTLV-1.

## Clinical vaccine trials

A safe and effective vaccine preparation in animal studies will eventually be considered for clinical trials. This would include phase 1 safety and pharmacokinetics studies in volunteers. Phase 2 and 3 trials would then be targeted to a population at risk of acquisition of HTLV-1. The largest and most suitable population would likely be individuals at significant risk of sexual acquisition of HTLV-1 in an endemic region.

## Conclusions

Previous studies in rabbits and monkeys suggest that inoculation with HTLV-1 gene products may provide protection against infection. The ideal vaccine candidate and method of inoculation remains to be deciphered. In addition, immune correlates of response remain to be determined. Although some studies suggest that neutralizing antibodies against the HTLV-1 envelope protein may provide protection against infection, the role of cytotoxic T lymphocyte responses against envelope and other viral proteins remains to be fully characterized. In addition, antibody-dependent cellular cytotoxicity, known to occur in primary infection (88–90), could also be important in vaccine protection.

The WHO issued a technical report in 2020 with a strong recommendation for global strategies to eliminate transmission of HTLV-1 (9). Thus, further studies of possible efficacy and safety of HTLV-1 vaccines is warranted. It will be interesting to determine why some people who are infected with HTLV-1, manage to maintain a very low proviral load set-point, and have a very low risk of disease. Analysis of such HTLV-1 “elite controllers” could provide important details to defining a protective response to the virus. If a safe and effective vaccine can be developed, it remains unclear which individuals might benefit from its use. Individuals at greatest risk of acquisition of HTLV-1, include people who are sexual partners of HTLV-1 infected subjects. In addition, a vaccine could have benefits in preventing maternal-to-child transfer. Although control of breast-feeding was effective in Japan in preventing vertical transmission, limiting breast-feeding in developing countries might cause malnutrition issues with newborns, so vaccination might have unique advantages in these settings. Thus, the highest prevalence of such individuals will be sexually active individuals in HTLV-1 endemic areas.

## Author contributions

The author confirms being the sole contributor of this work and has approved it for publication.

## Funding

This work was funded by PHS grants CA 252869, CA255095, CA100730, and AI156014 to LR.

## Conflict of interest

The author declares that the research was conducted in the absence of any commercial or financial relationships that could be construed as a potential conflict of interest.

## Publisher’s note

All claims expressed in this article are solely those of the authors and do not necessarily represent those of their affiliated organizations, or those of the publisher, the editors and the reviewers. Any product that may be evaluated in this article, or claim that may be made by its manufacturer, is not guaranteed or endorsed by the publisher.

## References

[1] Gessain A Cassar O. Epidemiological aspects and world distribution of HTLV-1 infection. Front Microbiol (2012) 3:article 388. doi: 10.3389/fmicb.2012.00388 23162541 PMC3498738

[2] Einsiedel L Pham H Talukder MR Taylor K Wilson K Kaldor J. Very high prevalence of infection with the human T cell leukaemia virus type 1c in remote Australian aboriginal communities: Results of a large cross-sectional community survey. PloS Negl Tropic Dis (2021) 15:e00099915. doi: 10.1371/journal.pntd.0009915 PMC8654171 34879069

[3] Cook LBM Taylor GP. HTLV-1 and HTLV-2 prevalence in the united states. J Infect Dis (2014) 209:486–7. doi: 10.1093/infdis/jit558 24154735

[4] Afonso PV Cassar O Gessain A. Molecular epidemiology, genetic variability and evolution of HTLV-1 with special emphasis on African genotypes. Retrovirology (2019) 16:39. doi: 10.1186/s12977-019-0504-z 31842895 PMC6916231

[5] Proietti FA Carneiro-Proietti ABF Catalan-Soares BC Murphy EL. Global epidemiology of HTLV-I and associated diseases. Oncogene (2005) 24:6058–68. doi: 10.1038/sj.onc.1208968 16155612

[6] Einsiedel L Pham H Wilson K Walley R Turpin J Bangham C. Human T-lymphotropic virus type 1c subtype proviral loads, chronic lung disease and survival in a prospective cohort of indigenous australians. PloS Negl Tropic Dis (2018) 12:e0006281. doi: 10.1371/journal.pntd.0006281 PMC5874075 29529032

[7] Paine E Garcia J Philpott TC Shaw G Ratner L. Limited sequence variation in human T-lymphotropic virus type 1 isolates from north American and African patients. Virology (1991) 182:111–23. doi: 10.1016/0042-6822(91)90654-T 2024459

[8] Hirons A Khoury G Purcell DFJ. Human T-cell lymphotropic virus type-1: A lifelong, persistent infection, yet never truly silent. Lancet Infect Dis (2021) 21:e2–e10. doi: 10.1016/S1473-3099(20)30328-5 32986997

[9] Bajis S Bull R Causer L Kaldor J Legrand N Martinello M. "Human T-lymphotropic virus type 1: Technical report. ISBN: 978-92-4-002022-1". Geneva, Switzerland: World Health Organization (2020).

[10] Goncalves DU Prioietti FA Ribas JGR Araujo MG Pinheiro SR Guedes AC. Epidemiology, treatment, and prevention of human T-cell leukemia virus type 1-associated diseases. Clin Microbiol Rev (2010) 23:577–89. doi: 10.1128/CMR.00063-09 PMC2901658 20610824

[11] Rosadas C Menezes MLB Galvao-Castro B Assone T Miranda AE Aragon MG. Blocking HTLV-1/2 silent transmission in Brazil: current public health policies and proposal for additional strategies. PloS Negl Tropic Dis (2021) 15:e0009717. doi: 10.1371/journal.pntd.0009717 PMC8460035 34555019

[12] Halbrook M Gadoth A Shankar A Zheng HQ Campbell EM Hoff NA. Human T-cell lymphotropic virus type 1 transmission dynamics in rural villages in the democratic republic of the Congo with high nonhuman primate exposure. PloS Negl Tropic Dis (2021) 15:e0008923. doi: 10.1371/journal.pntd.0008923 PMC7872225 33507996

[13] Moriuchi H Masuzaki H Doi H Katamine S. Mother-to-child transmission of human T-cell lymphotropic virus type 1. Pediatr Infect Dis (2013) 32:175–7. doi: 10.1097/INF.0b013e31827efc39 23328821

[14] Tagaya Y Matsuoka M Gallo R. 40 years of the human T-cell leukemia virus: past, present, and future. F1000 Rsearch (2019) 8:228. doi: 10.12688/f1000research.17479.1 PMC6396841 30854194

[15] Cook LB Melamed A Demontis MA Laydon DJ Fox JM Tosswill JH. Rapid dissemination of human T-lymphotropic virus type 1 during primary infection in transplant recipients. Retrovirology (2016) 13:3. doi: 10.1186/s12977-015-0236-7 26745892 PMC4706667

[16] Gross C Thoma-Kress AK. Molecular mechanisms of HTLV-1 cell-to-cell transmission. Viruses (2016) 8:74. doi: 10.3390/v8030074 27005656 PMC4810264

[17] Vanprooyen N Gold H Andresen V Schwartz O Jones K Ruscetti F. Human T-cell leukemia virus type 1 p8 protein increases cellular conduits and virus transmission. Proc Natl Acad Sci (2010) 107:20738–43. doi: 10.1073/pnas.1009635107 PMC2996430 21076035

[18] Igakura T Stionchcombe JC Goon PK Taylor GP Weber JN Griffiths GM. Spread of HTLV-1 between lymphocytes by virus-induced polarization of the cytoskeleton. Science (2003) 299:1713–6. doi: 10.1126/science.1080115 12589003

[19] Pais-Correia AM Sachse M Guadagnini S Robbiati V Lasserre R Gout O. Biofilm-like extracellular viral assemblies mediate HTLV-1 cell-to-cell transmission at virological synapses. Nat Med (2010) 16:83–9. doi: 10.1038/nm.2065 20023636

[20] Jones KS Petrow-Sadowski C Huang YK Bertolette DC Ruscetti FW. Cell-free HTLV-1 infects dendritic cells leading to transmission and transformation of CD4+ T cells. Nat Med (2008) 14:429–36. doi: 10.1038/nm1745 18376405

[21] Ratner L. Molecular biology of T cell leukemia virus. Semin Diagnositic Pathol (2020) 37:104–9. doi: 10.1053/j.semdp.2019.04.003 PMC6801043 31103249

[22] Grossman WJ Kimata JT Wong FH Zutter M Ley TJ Ratner L. Development of leukemia in mice transgenic for the tax gene of human T-cell leukemia virus type I. Proc Natl Acad Sci (1995) 92:1057–61. doi: 10.1073/pnas.92.4.1057 PMC42636 7862633

[23] Hasegawa H Sawa H Lewis MJ Orba Y Sheehy N Yamamoto Y. Thymus-derived leukemia-lymphoma in mice transgenic for the tax gene of human T-lymphotropic virus type I. Nat Med (2006) 12:466–72. doi: 10.1038/nm1389 16550188

[24] Satou Y Yasunaga J Zhao T Yoshida M Miyazato P Takai K. HTLV-1 bZIP factor induces T-cell lymphoma and systemic inflammation *in vivo* . PloS Pathog (2011) 7:e1001274. doi: 10.1371/journal.ppat.1001274 21347344 PMC3037353

[25] Grassmann R Aboud M Jeang K-T. Molecular mechanisms of cellular transformation by HTLV-I tax. Oncogene (2005) 24:5976–85. doi: 10.1038/sj.onc.1208978 16155604

[26] Bangham C. Human T cell leukemia virus type 1: Persistence and pathogenesis. Annu Rev Immnol (2018) 36:43–71. doi: 10.1146/annurev-immunol-042617-053222 29144838

[27] Ma G Yasunaga J Matsuoka M. Multifaceted functions and roles of HBZ in HTLV-1 pathogenesis. Retrovirology (2016) 13:16. doi: 10.1186/s12977-016-0249-x 26979059 PMC4793531

[28] Jones KS Lambert S Bouttieer M Benit L Ruscetti FW Hermine O. Molecular aspects of HTLV-1 entry: functional domains of the HTLV-1 surface subunit (SU) and their relationships to the entry receptors. Viruses (2011) 3:794–810. doi: 10.3390/v3060794 21994754 PMC3185769

[29] Li KS Zhang M Kronqvist M Wallin M Ekstrom M Derse D. Intersubunit disulfide isomerization controls membrane fusion of human T-cell leukemia virus. J Virol (2008) 82:7135–43. doi: 10.1128/JVI.00448-08 PMC2446982 18480461

[30] Kim FJ Manel N Garrido EN Valle C Sitbon M Battini JL. HTLV-1 and -2 envelope SU subdomians and critical determinants in receptor binding. Retrovirology (2004) 1:41. doi: 10.1186/1742-4690-1-41 15575958 PMC539286

[31] Rey FA Lok S-M. Common features of enveloped viruses and implications for immunogen design for next-generation vaccines. Cell (2018) 172:1319–34. doi: 10.1016/j.cell.2018.02.054 PMC7112304 29522750

[32] Monro JB Gorman J Ma X Zhou Z Arthos J Burton DR. Conformational dynamics of single HIV-1 envelope trimers on the surface of native virions. Science (2014) 346:759–63. doi: 10.1126/science.1254426 PMC4304640 25298114

[33] Nagy K Clapham P Cheingsong-Popov R Weiss RA. Human T-cell leukemia virus type: Induction of syncytia and inhibition by patients' sera. Int J Cancer (1983) 32:321–8. doi: 10.1002/ijc.2910320310 6604033

[34] Clapham P Nagy K Weiss RA. Pseudotypes of human T-cell leukemia virus types 1 and 2: neutralization by patients' sera. Proc Natl Acad Sci (1984) 81:2886–9. doi: 10.1073/pnas.81.9.2886 PMC345177 6326149

[35] Tanaka Y Zeng L Shiraki H Shida H Tozawa H. Identification of a neutralization epitope of the envelope gp46 antigen of human T cell leukemia virus type I and induction of neutralizing antibody by peptide immunization. J Immunol (1991) 147:364–0. 1711082

[36] Tanaka Y Tanaka R Terada E Koyanagi Y Miyano-Kurosaki N Yamamoto N. Induction of antibody responses that neutralize human T-cell leukemia virus type I infection *in vitro* and *in vivo* by peptide immunization. J Virol (1994) 68:6323–31. doi: 10.1128/jvi.68.10.6323-6331.1994 PMC237053 8083972

[37] Hadlock G Rowe J Perkins S Bradshaw P Song GY Cheng C. Neutralizing human monoclonal antibodies to conformational epitopes of human T-cell lymphotropic virus type 1 and 2 gp46. J Virol (1997) 71:5828–40. doi: 10.1128/jvi.71.8.5828-5840.1997 PMC191838 9223472

[38] Londos-Gagliardi D Armengaud MH Freund F Dalibart R Moze E Huet S. Antibodies directed against a variable and neutralizable region of the HTLV-I envelope surface glycoprotein. Leukemia (1997) 11(Suppl 3):38–41. 9209290

[39] Astier-Gin T Portail JP Londos-Gagliardi D Moynet D Blanchard S Dalibart R. Neutralizing activity and antibody reactivity toward immunogenic regions of the human T cell leukemia virus type 1 surface glycoprotein in sera of infected patients with different clinical states. J Infect Dis (1999) 175:716–9. doi: 10.1093/infdis/175.3.716 9041352

[40] Hadlock KG Rowe J Foung SK. The humoral immune response to human T-cell lymphotropic virus type 1 envelope glycoprotein gp46 is directed primarily against conformational epitopes. J Virol (1999) 73:1205–12. doi: 10.1128/JVI.73.2.1205-1212.1999 PMC103941 9882322

[41] Kuo C-WS Mirsaliotis A Brighty DW. Antibodies to the envelope glycoprotein of human T cell leukemia virus type 1 robustly activated cell-mediated cytotoxic response and directly neutralize viral infectivity at multiple steps of the entry process. J Immunol (2011) 187:361–71. doi: 10.4049/jimmunol.1100070 21646298

[42] Baba E Nakamura M Tanaka Y Kuroki M Itoyama Y Nakano S. Multiple neutralizing b-cell epitopes of human T-cell leukemia virus type 1 (HTLV-1) identified by human monoclonal antibodies. a basis for the design of an HTLV-1 peptide vaccine. J Immunol (1993) 151:1013–24. 7687611

[43] Takehara N Iwahara Y Uemura Y Sawada T Ohtsuki Y Iwai H. Effect of immunization on HTLV-I infection in rabbits. Int J Cancer (1989) 44:332–6. doi: 10.1002/ijc.2910440224 2759739

[44] Kataoka R Takehara N Iwahara Y Sawada T Ohtsuki Y Dawei Y. Transmission of HTLV-1 by blood transfusion and its prevention by passive immunization in rabbits. Blood (1990) 76:1657–61. doi: 10.1182/blood.V76.8.1657.1657 1976391

[45] Sawada T Iwahara Y Ishii K Taguchi H Hoshino H Miyoshi I. Immunoglobulin prophylaxis against milkborne transmission of human T cell leukemia virus type I in rabbits. J Infect Dis (1991) 164:1193–6. doi: 10.1093/infdis/164.6.1193 1955718

[46] Miyoshi I Takjehara N Sawada T Iwahara Y Kataoka R Yang D. Immunoglobulin prophylaxis against HTLV-I in a rabbit model. Leukemia 6(Supplement (1992) 1):24–6. 1347800

[47] Tanaka Y Ishii K Sawada T Ohtsuki Y Hoshino H Yanagihara R. Prophylaxis against a Melanesian variant of human T-lymphotropic virus type I (HTLV-I) in rabbits using HTLV-I immune globulin from asymptomiatcally infected Japanese carriers. Blood (1993) 82:3664–7. doi: 10.1182/blood.V82.12.3664.3664 8260703

[48] Bangham CR Osame M. Cellular immune response to HTLV-1. Oncogene (2005) 24:6035–46. doi: 10.1038/sj.onc.1208970 16155610

[49] Jeffery KJ Usuku K Hall SE Matsumoto W Taylor GP Procter J. HLA alleles determine human T-lymphotropic virus-I (HTLV-I) proviral load and the risk of HTLV-i-associated myelopathy. Proc Natl Acad Sci (1999) 96:3848–53. doi: 10.1073/pnas.96.7.3848 PMC22383 10097126

[50] Kubota R Nagai M Kawanishi T Osame M Jacobson S. Increased HTLV type 1 tax specific CD8+ cells in HTLV type 1-associated myelopathy/tropical spastic paraparesis: Correlation with HTLV type 1 proviral load. AIDS Res Hum Retroviruses (2000) 16:1705–9. doi: 10.1089/08892220050193182 11080814

[51] Vine AM Heaps AG Kaftantzi L Mosley A Asquith B Witkover A. The role of CTLs in persistent viral infection: cytolytic gene expression in CD8+ lymphocytes distinugishes between individuals with a high or low proviral load of human T cell lymphotropic virus type 1. J Immunol (2004) 173:5121–9. doi: 10.4049/jimmunol.173.8.5121 15470056

[52] Kubota R Hanada K Furukawa Y Arimura K Sosame M Gojobori T. Genetic stability of human T lymphotropic virus type I despite antiviral pressures by CTLs. J Immunol (2007) 178:5966–72. doi: 10.4049/jimmunol.178.9.5966 17442981

[53] Jacobson S Reuben JS Streilein RD Palker TJ. Induction of CD4+, human T lymphotropic virus type-1-specific cytotoxic T lymphomcytes from patients with HAM/TSP. Recognition of an immunogenic region of the gp46 envelop glycoprotein of human T lymphotropic virus type-1. J Immunol (1991) 146:1155–62. 1704032

[54] Kozako T Arima N Toji S Masamoto I Akimoto M Hamada H. Reduced frequency, diversity, and function of human T cell leukemia virus type 1-specific CD8+ T cell in adult T cell leukemia patients. J Immunol (2006) 177:5718–26. doi: 10.4049/jimmunol.177.8.5718 17015761

[55] Pique C Connan F Levilain JP Choppin J Dokhelar MC. Among all human T-cell leukemia virus type 1 proteins, tax, polymerase, and envelope proteins are predicted as preferential targets for the HLA-A2-restricted cytotoxic T-cell response. J Virol (1996) 70:4919–26. doi: 10.1128/jvi.70.8.4919-4926.1996 PMC190442 8763995

[56] Kozako T Akimoto M Toji S White Y Suzuki S Arima T. Target epitopes of HTLV-1 recognized by class I MHC-restricted cytotoxic T lymphocytes in patients with myelopathy and spastic paraparesis and infected patients with autoimmune disorders. J Med Virol (2011) 83:501–9. doi: 10.1002/jmv.21985 21264872

[57] Kattan T Macnamara A Rowan AG Nose H Mosley AJ Tanaka Y. The avidity and lytic efficiency of the CTL response to HTLV-1. J Immunol (2009) 182:5723–9. doi: 10.4049/jimmunol.0900069 19380819

[58] Zhimizu Y Takamori A Utunomiya A Kurimura M Yamano Y Hishizawa M. Impaired tax-specific T-cell responses with sufficient control of HTLV-1 in a subgroup of individuals at asymptomatic and smoldering stages. Cancer Sci (2009) 100:481–9. doi: 10.1111/j.1349-7006.2008.01054.x PMC11158518 19154412

[59] Mohsen MO Zha L Cabral-Miranda G Bachmann MF. Major findings and recent advances in virus-like particle (VLP)-based vaccines. Semin Immunol (2017) 34:123–32. doi: 10.1016/j.smim.2017.08.014 28887001

[60] Saxena M Vanderburg SH Melief CJM Bhardwaj N. Therapeutic cancer vaccines. Nat Rev Cancer (2021) 21:360–78. doi: 10.1038/s41568-021-00346-0 33907315

[61] Kosinska AD Bauer T Protzer U. Therapeutic vaccination for chronic hepatitis b. Curr Opin Virol (2017) 23:75–81. doi: 10.1016/j.coviro.2017.03.011 28453967

[62] Blass E Ott PA. Advances in the development of personalized neoantigen-based therapeutic cancer vaccines. Nat Rev Clin Oncol (2021) 18:215–29. doi: 10.1038/s41571-020-00460-2 PMC7816749 33473220

[63] Gray GE Bekker L-G Laher F Malahleha M Allen M Moodie Z. HVTN 702 study team. vaccine efficacy of ALVAC-HIV and bivalent subtype c gp120-MF59 in adults. New Engl J Med (2021) 384:1089–100. doi: 10.1056/NEJMoa2031499 PMC7888373 33761206

[64] Patel M Carritt K Lane J Jayappa H Stahl M Bourgeois M. Comparative efficacy of feline leukemia virus (FeLV) inactivated whole-virus vaccine and canarypox virus-vectored vaccine during virulent FeLV challenge and immunosuppression. Clin Vacine Immunol (2015) 22:798–805. doi: 10.1128/CVI.00034-15 PMC4478526 25972402

[65] Aida V Pliasas VC Neasham PJ North JF Mcwhorter KL Glover SR. Novel vaccine technologies in veterinary medicine: A herald to human medicine vaccines. Front Veterinary Sci (2021) 8:654289. doi: 10.3389/fvets.2021.654289 PMC8083957 33937377

[66] Gutierrez G Rodriguez SM Debrogniez A Gillet N Golime R Burny A. Vaccination against delta-retroviruses: The bovine leukemia virus paradigm. Viruses (2014) 6:2416–27. doi: 10.3390/v6062416 PMC4074934 24956179

[67] Abdala A Alvarez I Brossel H Calvinho L Carignano H Franco L. BLV: lessons on vaccine development. Retrovirology (2019) 16:26. doi: 10.1186/s12977-019-0488-8 31590667 PMC6781361

[68] Kazanji M Heraud J-M Merien F Pique C Dethe G Gessain A. Chimeric peptide vaccine composed of b- and T-cell epitopes of human T-cell leukemia virus type 1 induces humoral and cellular immune responses and reduces the proviral load in immunized squirrel monkeys (Saimiri sciureus). J Gen Virol (2006) 87:1331–7. doi: 10.1099/vir.0.81582-0 16603536

[69] Franchini G Tartaglia J Markham P Benson J Fullen J Wills M. Highly attenuated HTLV type I env poxvirus vaccines induce protection against a cell-associated HTLV type I challenge in rabbits. AIDS Res Hum Retrovirus (1995) 11:307–13. doi: 10.1089/aid.1995.11.307 7742044

[70] Hakoda E Machida H Tanaka Y Morishita N Sawada T Shida H. Vaccination of rabbits with recombinant vaccinia virus carrying the envelope gene of human T-cell lymphotropic virus type 1. Int J Cancer (1995) 60:567–70. doi: 10.1002/ijc.2910600423 7829272

[71] Nakamura H Hayami M Ohta Y Ishikawa K-I Tsujimoto H Kiyokawa T. Protection of cynomolgus monkeys against infection by human T-cell leukemia virus type-I by immunization with viral env gene products producted in escherichia coli. Int J Cancer (1987) 40:403–7. doi: 10.1002/ijc.2910400320 2887518

[72] Ibuki K Funahashi S-I Yamamoto H Nakamura M Igarashi T Miura T. Long-term persistence of protective immunity in cynomolgus monkeys immunized with a recombinant vaccinia virus expressing the human T cell leukaemia virus type I envelope gene. J Gen Virol (1997) 78:147–52. doi: 10.1099/0022-1317-78-1-147 9010298

[73] Kazanji M Tartaglia J Franchini G Dethoisy B Talarmin A Contamin H. Immunogenicity and protective efficacy of recombinant human T-cell leukemia/lymphoma virus type 1 NYVAC and naked DNA vaccine candidates in squirrel monkeys (Saimiri sciureus). J Virol (2001) 75:5939–48. doi: 10.1128/JVI.75.13.5939-5948.2001 PMC114309 11390595

[74] Grange MP Armand MA Audoly G Thollot D Desgranges C. Induction of neutralizing antibodies agianst HTLV-I envelope proteins after combined genetic and protein immunizations in mice. DNA Cell Biol (1997) 16:1439–48. doi: 10.1089/dna.1997.16.1439 9428792

[75] Armand MA Grange MP Paulin D Desgranges C. Targeted expression of HTLV-I envelope proteins in muscle by DNA immunization of mice. Vaccine (2000) 18:2212–22. doi: 10.1016/S0264-410X(99)00565-4 10717340

[76] Kabiri M Sankian M Sadri K Tafaghodi M. Robust mucosal and systemic responses against HTLV-1 by delivery of multi-epitope vaccine in PLGA nanoparticles. Eur J Phamaceutics Biopharmaceutics (2018) 133:321–30. doi: 10.1016/j.ejpb.2018.11.003 30408519

[77] Wijesundara DK Wodhiran N Cooney J O'donnell JS Avumegah MS Jaberolansar N. "Preclinical evaluation of a subunit vaccine platform for HTLV-1". In: International virtual conference on human retrovirology: HTLV 2022. Melbourne, Australia: Australian Government Department of Health (2022).

[78] Mcmillan CLD Cheung STM Modhiran N Barnes J Amarilla AA Bielefeldt-Ohmann H. Development of molecular clamp stablized hemagglutinin vaccines for influenza a viruses. NPJ Vaccines (2021) 6:135. doi: 10.1038/s41541-021-00395-4 34750396 PMC8575991

[79] Mulherkar R Karabudak A Ginwala R Huang X Rowan A Philip R. *In vivo* and *in vitro* immunogenciity of novel MHC class I presented epitopes to confer protective immunity against chronic HTLV-1 infection. Vaccines (2019) 36:5046–57. doi: 10.1016/j.vaccine.2018.07.002 PMC6091894 30005946

[80] Suehiro Y Hasegawa A Iino T Sasada A Watanabe N Matsuoka M. Clinical outcomes of a novel therapeutic vaccine with tax peptide-pulsed dendritic cells for adult T cell leukaemia/lymphoma in a pilot study. Br J Haematol (2015) 169:356–7. doi: 10.1111/bjh.13302 25612920

[81] Kannagi M Hasegawa A Nagano Y Iino T Okamura J Suehiro Y. Maintenance of long remission in adult T-cell leukemia by tax-targeted vaccine: a hope for disease-preventive therapy. Cancer Sci (2019) 110:849–57. doi: 10.1111/cas.13948 PMC6398881 30666755

[82] Ishizawa M Ganbaatar U Hasegawa A Takatsuka N Kondo N Yoneda T. Short-term cultured autologous peripheral blood mononuclear cells as a potential immunogen to activate tax-specific CTL response in adult T-cell leukemia patients. Cancer Sci (2021) 112:1161–72. doi: 10.1111/cas.14800 PMC7935807 33410215

[83] Kurihara K Harashima N Hanabuchi S Masuda M Utsunomiya A Tanosaki R. Potential immunogenicity of adult T cell leukemia cells *in vivo* . Int J Cancer (2005) 114:257–67. doi: 10.1002/ijc.20737 15551352

[84] Macnamara A Rowan A Hilburn S Kadolsky U Fujiwara H Suemori K. HLA class I binding of HBZ determines outcome in HTLV-1 infection. PloS Pathog (2010) 6:e1001117. doi: 10.1371/journal.ppat.1001117 20886101 PMC2944806

[85] Sugata K Yasunaga J Mitobe Y Miura M Miyazato P Kohara M. Protective effect of cytotoxic T lymphocytes targeting HTLV-1 bZIP factor. Blood (2015) 126:1095–105. doi: 10.1182/blood-2015-04-641118 26063164

[86] Jackson N Kester KE Casimiro D Gurunathan S Derosa F. The promise of mRNA vaccines: a biotech and industrial perspective. Vaccines (2020) 5:11. 32047656 10.1038/s41541-020-0159-8 PMC7000814

[87] Kisby T Yilmazer A Kostarelos K. Reasons for success and lessons learnt from nanoscale vaccines against COVID-19. Nat Nanotechnol (2021) 16:843–50. doi: 10.1038/s41565-021-00946-9 34381200

[88] Miyakoshi H Koide H Aoki T. *In vitro* antibody-dependent cellullar cytotoxicity against human T-cell leukemia/lymphoma virus (HTLV)-producing cells. Intenational J Cancer (1984) 33:287–91. doi: 10.1002/ijc.2910330302 6321358

[89] Sinclair AL Habeshaw JA Muir L Chandler P Forster S Cruickshank K. Antibody-dependent cell-mediated cytotoxicity: Comparison between HTLV-I and HIV-1 assays. AIDS (1988) 2:465–72. doi: 10.1097/00002030-198812000-00009 3149493

[90] Zhang XQ Yang L Ho DD Kuritzkes DR Chen IS Ching WT. Human T lymphotropic virus types I- and II-specific antibody-dependent cellular cytotoxicity: Strain specificity and epitope mapping. J Infect Dis (1992) 165:805–12. doi: 10.1093/infdis/165.5.805 1373751

